# Iatrogenic vs. Spontaneous Preterm Birth: A Retrospective Study of Neonatal Outcome Among Very Preterm Infants

**DOI:** 10.3389/fneur.2021.649749

**Published:** 2021-03-23

**Authors:** Xi Chen, Xiaoli Zhang, Wenhua Li, Wendong Li, Yong Wang, Shan Zhang, Changlian Zhu

**Affiliations:** ^1^Henan Key Laboratory of Child Brain Injury and Henan Pediatric Clinical Research Center, Institute of Neuroscience and Third Affiliated Hospital of Zhengzhou University, Zhengzhou, China; ^2^Center for Brain Repair and Rehabilitation, Institute of Neuroscience and Physiology, Sahlgrenska Academy, University of Gothenburg, Gothenburg, Sweden; ^3^Department of Women's and Children's Health, Karolinska Institutet, Stockholm, Sweden

**Keywords:** preterm birth, spontaneous preterm birth, iatrogenic preterm birth, neonatal outcome, neonatal complication

## Abstract

**Objective:** Preterm birth is a leading contributor to childhood morbidity and mortality, and the incidence tends to increase and is higher in developing countries. The aim of this study was to analyze the potential impact of preterm birth in different etiology groups on neonatal complications and outcomes and to gain insight into preventive strategies.

**Methods:** We performed a retrospective cohort study of preterm infants less than 32 weeks' gestation in the Third Affiliated Hospital of Zhengzhou University from 2014 to 2019. Preterm births were categorized as spontaneous or iatrogenic, and these groups were compared for maternal and neonatal characteristics, neonatal complications, and outcomes. All infants surviving at discharge were followed up at 12 months of corrected age to compare the neurodevelopmental outcomes.

**Results:** A total of 1,415 mothers and 1,689 neonates were included, and the preterm population consisted of 1,038 spontaneous preterm infants and 651 iatrogenic preterm infants. There was a significant difference in the incidence of small for gestational age between the two groups. Infants born following spontaneous labor presented with a higher risk of intraventricular hemorrhage, whereas iatrogenic preterm birth was associated with higher risk of necrotizing enterocolitis and coagulopathy and higher risk of pathoglycemia. There was no difference in mortality between the two groups. Follow-up data were available for 1,114 infants, and no differences in neurologic outcomes were observed between the two preterm birth subtypes.

**Conclusions:** Preterm births with different etiologies were associated with some neonatal complications, but not with neurodevelopmental outcomes at 12 months of corrected age.

## Introduction

Preterm birth is the leading cause of neonatal mortality and morbidity and is a major risk factor of neurodevelopmental impairment (1, 2). It is estimated that preterm birth occurs in 11% of all worldwide deliveries (3). Even though neonatal intensive care has improved in recent years, there is still a wide range of neurodevelopmental disabilities resulting from very preterm birth (2, 4). Thus, preterm infants and especially very preterm infants remain a challenging problem within the field of perinatology.

Preterm birth can result from many possible etiologies, but the two major clinical etiologies of preterm birth are iatrogenic and spontaneous preterm birth (5). Iatrogenic preterm birth, including labor induction and cesarean delivery without labor, constitutes about 30–40% of all preterm births, and pre-eclampsia/eclampsia and severe intrauterine growth restriction are the common causes (6). Spontaneous preterm birth can result from multiple causes such as infection or inflammation, cervical factors, hemorrhage, decidual senescence, stress, genetics, and sociodemographic factors (7, 8).

Gestational age (GA) at birth is the strongest predictor of neonatal complications and outcomes (9), and all preterm infants presumably experience some risk due to immaturity. In addition, some causes of preterm delivery may be dangerous in themselves beyond the effects related to immaturity. Some studies have investigated the differences between etiologies of preterm birth that might affect neonatal outcomes, for example, iatrogenic preterm birth has been shown to increase neonatal mortality and severe morbidity and to result in worse psychomotor outcomes compared with spontaneous preterm birth (10, 11).

There is a growing awareness of the role of pathologies in preterm mortality and morbidity. Given the heterogeneity within clinical etiologies of preterm birth, it is helpful to examine the etiologies separately. In addition, identifying the specific characteristics of each clinical etiology is essential for developing effective methods for preventing preterm birth (12). There are limited data, however, regarding how the indications for premature delivery affect the infant's outcomes. Thus, the aim of this study was to investigate if there is any link between etiologies of very preterm birth and neurodevelopmental outcomes.

## Methods

### Study Population

This was a retrospective, hospital-based cohort study carried out at the Third Affiliated Hospital of Zhengzhou University between 1 July 2014 and 30 June 2019. This study included all preterm infants born in this hospital with a GA < 32 weeks. Infants delivered at other hospitals and transferred to the NICU at the Third Affiliated Hospital of Zhengzhou University within 7 days after birth were also included if the detailed pregnancy and delivery records were available. Exclusion criteria included stillbirths, delivery room deaths, malformed fetuses, and infants for whom the pregnancy information was not fully recorded. This study was approved by the ethics committee of Third Affiliated Hospital of Zhengzhou University and was exempt from the requirement for informed consent.

### Definition and Data Collection

Newborn infants at GA < 32 weeks were included. The study cohort was classified into spontaneous preterm delivery (due to preterm labor with regular uterine contractions and cervical changes or with preterm rupture of membranes) and iatrogenic preterm delivery (due to any maternal or fetal medical complication necessitating early delivery) (13). Indications for maternal admission and delivery were identified from the hospital admission and delivery records. Clinical information, demographic data, gestation and delivery characteristics, and major neonatal conditions were collected. GA was established based on first-trimester ultrasound estimations in the majority of patients and in a few patients by the last menstrual bleeding before pregnancy. The following parameters were included in the analysis: small for gestational age (SGA, defined as birth weight < 10th percentile for gestational age), asphyxia (14), bronchopulmonary dysplasia (BPD, defined simply by considering the need for oxygen at 28 days after birth) (15), pneumonia, anemia (16), patent ductus arteriosus (17), persistent pulmonary hypertension (18), coagulopathy (19), retinopathy of prematurity (20), necrotizing enterocolitis (NEC, Bell's stage ≥ II) (16), intraventricular hemorrhage (IVH, diagnosed by intracranial ultrasound and graded from 1 to 4 on the Papile scale) (21), periventricular leukomalacia (PVL, diagnosed by intracranial ultrasound or brain magnetic resonance imaging) (22), pathoglycemia (blood sugar < 2.2 mmol/L or > 7 mmol/L), and neonatal death (died before 28 days of life).

### Follow-Up

All survivors were assessed at 3, 6, and 12 months of corrected age by trained specialists. Development was evaluated using the Bayley Scales of Infant and Toddler Development-Chinese version, including the psychomotor developmental index and the mental developmental index. Developmental delay was indicated by scores <70 on either scale. The criteria for the diagnosis of cerebral palsy included permanent movement and posture disorders caused by non-progressive disturbances that occurred in the developing fetal or neonatal brain (23). Adverse neurological outcomes included cerebral palsy, blindness (corrected visual acuity <0.05), deafness (hearing loss requiring amplification or worse), and development delay.

### Statistical Analysis

Data were analyzed using the SPSS 26.0 software (IBM, Armonk, NY). Descriptive statistics are presented using absolute and relative frequencies for categorical variables, and continuous variables are presented as means ± standard deviations. Categorical data were compared using either Fisher's Exact Test or the chi-square test, and continuous data were analyzed with ANOVA for normally distributed variables and by Kruskal–Wallis tests for non-normally distributed variables. The multivariate analysis was adjusted for GA, birthweight, gender, and delivery model, with the reference group being spontaneous labor onset. All tests were two-tailed, and *p* < 0.05 was considered statistically significant.

## Results

### Neonatal Characteristics of Preterm Birth

The study population consisted of 1,415 mothers and 1,689 neonates. Among the 1,689 neonates, 1,122 were singletons and 567 were twins or triplets (of which 203 were with assisted reproductive technology). The preterm infants were classified as spontaneous (*n* = 1,038; 61.5%) or iatrogenic (*n* = 651; 38.5%). The ratio of gender, incidence of asphyxia, percentage of erythropoietin treatment, and duration of mechanical ventilation showed no differences between the two groups. GA and birth weight were significantly different between the groups, with the iatrogenic group having the higher GA and lower birth weight. The overall prevalence of SGA was 10.0%, which was much higher in the iatrogenic group than that in the spontaneous group (21.7 vs. 2.7%, *p* < 0.001). In addition, the cesarean section rate in the iatrogenic group was more than twice that in the spontaneous group (94.8 vs. 46.3%, *p* < 0.001), which indicates that vaginal delivery is not an option for most iatrogenic very preterm infants (Table 1).

**Table 1:** Neonatal characteristics by preterm birth subtype.

	**Spontaneous (*n* = 1,038)**	**Iatrogenic (*n* = 651)**	**Total (*n* = 1,689)**	***P*-value**
**Gestational age, w**	29.5 ± 1.5	30.1 ± 1.3	29.7 ± 1.5	0.000
<28 w, *n* (%)	152 (14.6%)	34 (5.2%)	186 (11.0%)	0.000
28–29^+6^ w, *n* (%)	419 (40.4%)	226 (34.7%)	645 (38.2%)	0.020
30–31^+6^ w, *n* (%)	467 (45.0%)	391 (60.1%)	858 (50.8%)	0.000
**Gender, male**, *n* (%)	600 (57.8%)	369 (56.7%)	969 (57.4%)	0.650
**Birth weight, g**	1,347 ± 291	1,240 ± 289	1,306 ± 295	0.000
<1,000, *n* (%)	108 (10.4%)	115 (17.7%)	223 (13.2%)	0.000
1,000–1,500, *n* (%)	673 (64.8%)	443 (68.0%)	1,116 (66.1%)	0.175
>1,500, *n* (%)	257 (24.8%)	93 (14.3%)	350 (20.7%)	0.000
**Cesarean**, ***n*** **(%)**	481 (46.3%)	617 (94.8%)	1,098 (65.0%)	0.000
**Asphyxia**				0.355
None	591 (56.9%)	350 (53.8%)	941 (55.7%)	0.201
Mild	406 (39.1%)	269 (41.3%)	675 (40.0%)	0.367
Severe	41 (3.9%)	32 (4.9%)	73 (4.3%)	0.342
**Days in hospital**	33.7 ± 20.7	38.4 ± 21.1	35.5 ± 21.0	0.000
**SGA**	28 (2.7%)	141 (21.7%)	169 (10.0%)	0.000
**EPO therapy**, ***n*** **(%)**	421 (40.6%)	293 (45.0%)	714 (42.3%)	0.072
**Transfusions**, ***n***	1.83 ± 2.0	2.18 ± 2.3	1.97 ± 2.1	0.001
**PS usage, times**				0.003
0, *n* (%)	87 (8.4%)	42 (6.5%)	129 (7.6%)	0.146
1, *n* (%)	762 (73.4%)	448 (68.8%)	1,210 (71.6%)	0.042
≥2, *n* (%)	189 (18.2%)	161 (24.7%)	350 (20.7%)	0.001
**Mechanical ventilation, days**				0.339
0, *n* (%)	197 (19.0%)	106 (16.3%)	303 (17.9%)	0.160
≤ 7, *n* (%)	532 (51.3%)	351 (53.9%)	883 (52.3%)	0.286
>7, *n* (%)	309 (29.8%)	194 (29.8%)	503 (29.8%)	0.989

*SGA, small for gestational age; EPO, erythropoietin; PS, pulmonary surfactant*.

### Maternal Characteristics of Preterm Birth

Women who underwent an iatrogenic preterm delivery tended to be older than the spontaneous group (*p* < 0.001). There were also relatively more multiple gestations in the spontaneous group (27.4%) compared to the iatrogenic group (11.3%). As expected, hypertensive disorder was the most common indication for early pregnancy termination in the iatrogenic group (72.8%), followed by fetal distress (40.9%) and placental abruption (11.8%). Meanwhile, women who underwent a spontaneous preterm delivery were more likely to have received antibiotics (10 vs. 3.0%) compared to the iatrogenic group for the treatment of ureaplasma urealyticum infection, chorioamnionitis, and streptococcal infection. The use of magnesium sulfate to prevent eclampsia was higher in the iatrogenic group (21.8%) than in the spontaneous group (9.6%; Table 2).

**Table 2:** Maternal characteristics by preterm birth subtype.

	**Spontaneous (*n* = 824)**	**Iatrogenic (*n* = 591)**	**Total (*n* = 1,415)**	***P*-value**
**Maternal age, years**	30.0 ± 4.8	31.7 ± 5.5	30.7 ± 5.2	0.000
**Maternal age**, **≥35 years**	155 (18.8%)	181 (30.6%)	336 (23.7%)	0.000
**Nulliparous**, ***n*** **(%)**	414 (50.2%)	237 (40.1%)	651 (46.0%)	0.000
**Multiple gestation**, ***n*** **(%)**	226 (27.4%)	67 (11.3%)	293 (20.7%)	0.000
**Previous preterm birth**, ***n*** **(%)**	169 (20.5%)	119 (20.1%)	288 (20.4%)	0.863
**Previous miscarriages**				0.000
0, *n* (%)	455 (55.2%)	266 (45.0%)	721 (51.0%)	0.000
1–2, *n* (%)	315 (38.2%)	273 (46.2%)	588 (41.6%)	0.003
≥3, *n* (%)	54 (6.6%)	52 (8.8%)	106 (7.5%)	0.114
**Medical treatment**				
Maternal antibiotics, *n* (%)	82 (10.0%)	18 (3.0%)	100 (7.1%)	0.000
Magnesium sulfate, *n* (%)	78 (9.6%)	129 (21.8%)	208 (14.7%)	0.000
Antenatal corticosteroids, *n* (%)	625 (75.8%)	477 (80.7%)	1,102 (77.9%)	0.030
**Maternal complications**				
Meconium-stained fluid, *n* (%)	36 (4.4%)	30 (5.1%)	66 (4.7%)	0.534
Fetal distress, *n* (%)	89 (10.8%)	241 (40.8%)	330 (23.3%)	0.000
Placental abruption, *n* (%)	44 (5.3%)	70 (11.8%)	114 (8.1%)	0.000
Gestational diabetes, *n* (%)	74 (9.0%)	61 (10.3%)	135 (9.5%)	0.397
Hypertensive disorders, *n* (%)	24 (2.9%)	430 (72.8%)	454 (32.1%)	0.000

### Neonatal Complications

The most common complication in this study cohort was respiratory distress syndrome, but there was no significant difference between the two groups. A greater frequency of NEC (6.5 vs. 2.7%, *p* < 0.001) and coagulopathy (18.1 vs. 11.8%, *p* < 0.001) was observed in the iatrogenic group, while the spontaneous group had a greater frequency of IVH (35.0 vs. 29.6%, *p* = 0.023). In addition, the ratio of pathoglycemia was relatively low, but it occurred more frequently in the iatrogenic group (2.2 vs. 0.8%, *p* = 0.015) compared to the spontaneous group. There was no difference in neonatal mortality between the two groups for infants < 30 weeks. For infants born at 30–31^+6^ weeks' gestation, the risk of death was greater in the iatrogenic group than the spontaneous group (14.6 vs. 8.8%, *p* = 0.008; Table 3). Multivariate logistic regression analysis showed slight changes in neonatal complications (Table 4). Compared to the spontaneous group, the iatrogenic group was associated with higher risk of NEC [OR 2.629, *p* = 0.002], coagulopathy [OR 1.760, *p* = 0.001], and pathoglycemia [OR 3.750, *p* = 0.029].

**Table 3:** Neonatal short-term complications and mortality by preterm birth subtype.

	**Spontaneous (*n* = 1,038)**	**Iatrogenic (*n* = 651)**	**Total (*n* = 1,689)**	***P*-value**
RDS	986 (95.0%)	627 (96.3%)	1,613 (95.5%)	0.202
Pneumonia	657 (63.3%)	434 (66.7%)	1,091 (64.6%)	0.158
Severe anemia	587 (56.6%)	372 (57.1%)	959 (56.8%)	0.811
PPHN	247 (23.8%)	156 (24.0%)	403 (23.9%)	0.937
PDA	400 (38.5%)	250 (38.4%)	650 (38.5%)	0.956
NEC (II-III)	28 (2.7%)	42 (6.5%)	70 (4.1%)	0.000
ROP	22 (2.1%)	17 (2.6%)	39 (2.3%)	0.512
BPD	366 (35.3%)	234 (35.9%)	600 (35.5%)	0.775
Cholestasis	156 (15.0%)	100 (15.4%)	256 (15.2%)	0.853
IVH	363 (35.0%)	193 (29.6%)	556 (32.9%)	0.023
IVH(III-IV)	65 (6.3%)	25 (3.8%)	90 (5.3%)	0.031
Coagulopathy	123 (11.8%)	118 (18.1%)	241 (14.3%)	0.000
PVL	37 (3.6%)	19 (2.9%)	56 (3.3%)	0.471
Pathoglycemia	8 (0.8%)	14 (2.2%)	22 (1.3%)	0.015
In-hospital death	197 (19.0%)	128 (19.7%)	325 (19.2%)	0.729
<28w	73 (48.0%)	19 (55.9%)	92 (49.5%)	0.408
28–29^+6^ w	83 (19.8%)	52 (23.0%)	135 (20.9%)	0.341
30–31^+6^ w	41 (8.8%)	57 (14.6%)	98 (11.4%)	0.008

*RDS, respiratory distress syndrome; PPHN, persistent pulmonary hypertension; PDA, patent ductus arteriosus; NEC, necrotizing enterocolitis; ROP, retinopathy of prematurity; BPD, bronchopulmonary dysplasia; IVH, intraventricular hemorrhage; PVL, periventricular leukomalacia*.

**Table 4:** Logistic regression analysis for neonatal complications and mortality.

	**Odds ratio**	**95% confidence interval**	***P*-value**
RDS	1.028	0.577–1.831	0.926
PPHN	1.162	0.881–1.533	0.289
PDA	1.060	0.830–1.353	0.641
NEC (II–III)	2.629	1.409–4.908	0.002
ROP	1.221	0.505–2.949	0.658
BPD	0.950	0.730–1.237	0.704
IVH (III–IV)	0.810	0.460–1.426	0.465
Coagulopathy	1.760	1.255–2.469	0.001
Pathoglycemia	3.750	1.144–12.291	0.029
Mortality	1.370	0.979–1.917	0.066

*Adjusted for gestational age, birthweight, delivery method, and fetal gender; RDS, respiratory distress syndrome; PPHN, persistent pulmonary hypertension; PDA, patent ductus arteriosus; NEC, necrotizing enterocolitis; ROP, retinopathy of prematurity; BPD, bronchopulmonary dysplasia; IVH, intraventricular hemorrhage*.

### Adverse Outcomes in Preterm Infants

The preterm infants were followed up to 12 months of corrected age. Twenty-two infants died (spontaneous group: 14; iatrogenic group: 8) during the follow-up period, and 250 infants were completely lost to follow-up (spontaneous group: 158; iatrogenic group: 92). Follow-up information was therefore available for 1,114 infants, and no differences in poor outcomes were observed between the two preterm birth subtypes either for GA < 28 weeks or for GA 28–31^+6^ weeks (Table 5).

**Table 5:** Outcomes comparison between both groups at 12 months of corrected age.

	**Spontaneous (*n* = 683)**	**Iatrogenic (*n* = 431)**	**OR [95% CI]**	***P*-value**
**Death**, ***n*** **(%)**	14/683 (2.0%)	8/431 (1.9%)	0.90 [0.38–2.17]	0.821
<28 w	5/63 (7.9%)	1/13 (7.7%)	0.97 [0.10–9.04]	0.976
28–31^+6^ w	9/620 (1.5%)	7/418 (1.7%)	1.16 [0.43–3.13]	0.775
**Disability**, ***n*** **(%)**	60/669 (9.0%)	47/423 (11.1%)	1.27 [0.85–1.90]	0.246
<28 w	5/58 (8.6%)	1/12 (8.3%)	0.96 [0.10–9.08]	0.974
28–31^+6^ w	55/611 (9.0%)	46/411 (11.2%)	1.27 [0.84–1.93]	0.250
Cerebral palsy, *n* (%)	13/669 (1.9%)	9/423 (2.1%)	1.10 [0.47–2.59]	0.833
Blindness, *n* (%)	9/669 (1.3%)	4/423 (0.9%)	0.70 [0.21–2.29]	0.553
Deafness, *n* (%)	7/669 (1.0%)	6/423 (1.4%)	1.36 [0.45–4.08]	0.581
Development delay, *n* (%)	47/669 (7.0%)	38/423 (9.0%)	1.31 [0.84–2.04]	0.239
**Death** **+** **disability**, ***n*** **(%**)	74/683 (10.8%)	55/431 (12.8%)	1.20 [0.83–1.75]	0.328
<28 w	10/63 (15.9%)	2/13 (15.4%)	0.96 [0.19–5.02]	0.965
28–31^+6^ w	64/620 (10.3%)	53/418 (12.7%)	1.26 [0.86–1.86]	0.239

## Discussion

Preterm delivery is one of the most important causes of perinatal morbidity and mortality. This retrospective study showed that iatrogenic preterm births with GA < 32 weeks were more vulnerable to some complications.

The rate of iatrogenic preterm birth in this study was slightly higher than that described previously (5), which might be due to the fact that preterm births related to medical indications are transferred to tertiary hospitals in China, and this is more common for pregnancy with GA < 32 weeks (24). Neonates in the iatrogenic group were more likely to be low-birth-weight with higher GA because the abnormal intrauterine environment was unfavorable for fetal growth, which is an indication for early pregnancy termination. Women facing increased risk of problems during pregnancy, including hypertension and placental abruption, are more likely to need cesarean section. The prevalence of SGA was higher in the iatrogenic group, which indicated that the fetus was more likely to be affected by pregnancy complications.

When comparing neonatal complications, 13 parameters were selected for descriptive analysis. Although there was no difference between the two groups regarding the incidence of pneumonia, the incidence was over 60% in both groups, which indicated that preterm neonates with GA < 32 weeks were more likely to have had pneumonia diagnosed. Lung inflammation in the neonate may be initiated by events that occur before birth or as a result of respiratory therapies used to treat lung disease. Premature infants are particularly vulnerable because they under-express protective factors like the anti-inflammatory cytokine IL-10 and Clara cell protein (25) and because they have immature immune systems with reduced innate and adaptive immunity, which can greatly affect their ability to fight infection (26). Significant differences were observed between the two groups in terms of the incidence of NEC. Some studies have demonstrated that fetal distress, preeclampsia, birthweight, and cesarean section are associated with NEC, and maternal preeclampsia in particular might be an important risk factor for the development of NEC in premature infants (27). Moreover, our results suggest that infants delivered early on the basis of medical indications might have an increased risk of coagulopathy, and preterm newborns are considered to be at risk of acquired coagulopathy (28). Thus, it appears that maternal complications might have an impact on the development of NEC and coagulopathy. IVH was a common complication among infants in the spontaneous group, which was consistent with prior reports (29, 30). There are several possible explanations for the outcomes of coagulopathy, NEC, and IVH. First, GA plays a vital role, and the GA at birth was slightly higher in the iatrogenic group compared to the spontaneous group. Second, the administration of antenatal corticosteroids (ACS) is the state-of-the-art for reducing adverse neonatal outcomes and can reduce the incidence of both PVL and severe IVH (31). Pregnant women in the iatrogenic group are often admitted to hospital early due to pregnancy symptoms. According to their condition, the doctor will use ACS to promote fetal lung maturation, and this allows them to choose the appropriate timing of pregnancy. In contrast, the spontaneous group has only a short window between admission and birth in which they can be treated with ACS. Third, hypertensive disorders were the major pregnancy complications experienced by the iatrogenic group in the present study, and this was consistent with a previous study (32). Antenatal magnesium sulfate is used in obstetrics as a first line anticonvulsant in the treatment of eclampsia and in preventing the progression of preeclampsia to eclampsia. A high frequency of the use of magnesium sulfate to prevent the occurrence of eclampsia was observed in the iatrogenic group, and some initial studies have suggested that magnesium sulfate may have a protective role against IVH in preterm neonates (33, 34). The finding in this study of an increased risk of pathoglycemia among infants born after iatrogenic preterm delivery is interesting. One study indicated that hypo- and hyperglycemia are common in preterm infants, especially SGA infants and those with low Apgar score (35). Similar to that study, we found that the prevalence of SGA was much higher in the iatrogenic group than in the spontaneous group, and this might explain the difference in the incidence of pathoglycemia we observed between the two groups. However, our study included only the preterm infants with GA < 32 weeks, which is different from other studies. The relevance of this difference appears to be questionable due to the small number of patients from a clinical point of view, and further research is needed.

Numerous studies have evaluated the relationship between preterm birth subtypes and neonatal death risk, but these have reported inconsistent results (29, 30, 36). Similar to prior studies, our study did not detect any significant differences in mortality rates. However, for infants born at 30–31^+6^ weeks' GA, the risk of death was greater in the iatrogenic group, which indicated that the underlying causes of iatrogenic preterm birth might be diverse at this GA. This reflects the fact that these infants were induced due to severe pregnancy complications, which might have increased their risk of death. For infants with lower GA, there were no differences between the two groups, and it is likely that the shorter GA diminished the significance of the differences in mortality between the groups. We also observed no significant difference in neurological outcome between the spontaneous group and the iatrogenic group. One explanation for this might be that only neonates still alive at follow-up were included in this study, and aggressive medical care was beneficial to the children's outcomes. Whether infants received rehabilitation therapy depended on parental attitudes and socio-economic factors. The small number of children with adverse neurological outcomes in the follow-up cohort, particularly children with cerebral palsy, might also explain this lack of difference between the two groups. In addition, the rate of infants lost to follow-up was not negligible. Therefore, conclusions should be drawn with caution because unknown confounding factors might have accounted for this observation.

Some limitations of this study should be noted. First, our results were restricted to very preterm infants (GA < 32 weeks) in a single maternity hospital, which might limit generalizability. Second, the rate of lost to follow-up was 18%, and it is possible that children with poor outcomes were not followed up, which might have biased the results. Finally, multiple factors are associated with the neurodevelopment outcomes, and not only preterm birth itself and its subtypes, but also medical care and socio-economic factors might affect the outcomes. Despite these limitations, this study has several strengths. This study chose to classify the etiologic types of preterm birth according to the reason for delivery by consulting obstetric records and collecting neonatology records, which ensured the accuracy of the data to the greatest extent possible. The assessment of both neonatal and infancy period outcomes in very preterm infants was another strength. This investigation might provide guidance for future research.

In conclusion, some neonatal complications were associated with etiologic types of very preterm birth. However, we evaluated only the neonatal and infancy period outcomes of the preterm infants in this study. Thus, further research is needed on the long term consequences of preterm birth subtype. Outcomes beyond survival are also important, especially long-term neurological, cognitive, and behavioral consequences. Finally, we strongly support the view that it is the understanding of the association between neonatal complications and preterm birth clinical subtypes that will pave the way to the prevention of adverse outcomes.

## Data Availability Statement

The original contributions presented in the study are included in the article/supplementary material, further inquiries can be directed to the corresponding author/s.

## Ethics Statement

The studies involving human participants were reviewed and approved by the ethics committee of Third Affiliated Hospital of Zhengzhou University. Written informed consent to participate in this study was provided by the participants' legal guardian/next of kin.

## Author Contributions

CZ designed the study. XC, XZ, WenhL, WendL, YW, and SZ were involved in data collection. XC and XZ analyzed the data and wrote the paper. All authors read and approved the final manuscript.

## Conflict of Interest

The authors declare that the research was conducted in the absence of any commercial or financial relationships that could be construed as a potential conflict of interest.
